# Soil management strategies shape bacterial and eukaryotic community structure in organic and inorganic systems of *Malus* × *domestica* production

**DOI:** 10.1038/s41598-026-49450-x

**Published:** 2026-04-30

**Authors:** Kamila Łucja Bokszczanin, Aleksandra Chojnacka, Marzena Suchocka, Hazem M. Kalaji, Ryszard Malinowski, Marcin Kubus

**Affiliations:** 1 Department of Pomology and Horticulture Economics, Institute of Horticultural Sciences, Warsaw University of Life Sciences-SGGW, Nowoursynowska 159 Str., 02-787 Warsaw, Poland; 2 Department of Biochemistry and Microbiology, Institute of Biology, Warsaw University of Life Sciences-SGGW, Nowoursynowska 159 Str., 02-787 Warsaw, Poland; 3 Department of Landscape Architecture, Institute of Environmental Engineering, Warsaw University of Life Sciences-SGGW, Warsaw, Poland; 4 Department of Plant Physiology, Institute of Biology, Warsaw, University of Life Sciences - SGGW, Nowoursynowska St. 159, 02-776 Warsaw, Poland; 5 Department of Environmental Management, West Pomeranian University of Technology in Szczecin, Słowackiego 17 Street, 71-434 Szczecin, Poland; 6 Department of Landscape Architecture, West Pomeranian University of Technology in Szczecin, Słowackiego 17 Street, 71-434 Szczecin, Poland

**Keywords:** Rhizosphere microbiome, Bacterial and fungal diversity, Orchard soil management, Mulching treatments, Ecology, Ecology, Environmental sciences, Microbiology, Plant sciences

## Abstract

Understanding the temporal dynamics of soil microbial communities is crucial for assessing the stability of orchard soils. We analyzed bacterial and eukaryotic communities in the rhizosphere of apple trees under seven mulching treatments across two growing seasons (2020 and 2023). To account for potential batch effects between separate sequencing runs, results from each year were juxtaposed to identify recurring microbial patterns. High-throughput 16S and ITS sequencing revealed that management-specific signatures persisted over time despite inter-run variability. Bacterial alpha diversity varied significantly among individual treatments, while beta diversity consistently distinguished management regimes across both years. Fungal alpha diversity remained largely unaffected, but beta diversity revealed significant compositional shifts between organic and inorganic groups. Organic mulching (shredded *Miscanthus* straw and spent mushroom compost) consistently enriched bacterial families linked to organic matter turnover (*Sphingomonadaceae*, *Flavobacteriaceae*, *Moraxellaceae*), whereas inorganic systems favored *Comamonadaceae* and *Nitrosomonadaceae*. Organic soils were also enriched in saprotrophic fungi (*Lasiosphaeriaceae*) and broader eukaryotes (*Ciliophora*), while inorganic – *Mortierellaceae*. Network analyses identified a cohesive bacterial core, whereas fungal/eukaryotic communities were more modular and responsive to mulching type. These findings demonstrate that sustained organic mulching promotes stable microbial configurations associated with soil functioning, highlighting the consistency of management-associated microbial signatures under long-term agricultural practices.

**Supplementary Information:**

The online version contains supplementary material available at 10.1038/s41598-026-49450-x.

## Introduction

Soil microbial communities play a crucial role in plant productivity, nutrient cycling, and ecosystem functioning, particularly in perennial cropping systems such as apple orchards. These communities are shaped by a complex interplay of environmental factors, plant–microbe interactions, and agronomic management practices. Soil degradation is increasingly reported in apple orchards, largely due to the absence of crop rotation and the long-term maintenance of herbicide strips, which prevent the incorporation of organic matter into the soil^1^. The resulting decline in humus content not only reduces soil fertility and nutrient availability but also negatively affects soil structure and overall ecosystem functioning^2^. Moreover, continuous apple cultivation on the same site predisposes orchards to apple replant disease (ARD), a complex disorder strongly associated with an increased abundance of pathogenic fungi, oomycetes, and plant-parasitic nematodes in the rhizosphere^3^.

To mitigate these problems, the adoption of soil management practices that enhance organic matter inputs is indispensable. Such practices enhance soil physicochemical properties, facilitate the release of mineral nutrients for plant uptake, and promote a more balanced and functional soil microbiome^3^. Given its multifunctional role in nutrient cycling, disease suppression, and plant growth promotion, the rhizosphere microbiome should be considered a central component in the design of sustainable orchard floor management strategies^4,5^.

While many studies have explored how different soil management strategies affect the composition and diversity of the rhizosphere microbiome, far less is known about the temporal dynamics of these communities under stable and long-term conditions^6,7^.

Temporal variation in microbial communities can arise from seasonal fluctuations, interannual climate variability, or stochastic processes. However, under consistent environmental and management conditions, it is reasonable to expect that a stable “core” microbiome might persist year to year, while more transient or responsive taxa may fluctuate. Understanding which components of the rhizosphere microbiome are stable versus variable over time is critical for predicting microbial contributions to plant health and for designing resilient agricultural systems^8^. Current research is increasingly focused on uncovering the functions of the microbiome and on promoting microbe-based strategies to boost crop yields and support sustainable food systems. This shift is highlighted by initiatives aimed at establishing model host–microbiome frameworks, identifying core microbiomes and metagenomes, and clarifying the principles behind assembling synthetic microbiomes. Key priorities involve dissecting the interactions among genotype, environment, microbiome, and management practices^9^. Effectively incorporating beneficial plant-associated microbiomes into agriculture demands a thorough understanding of these complex relationships within contemporary farming systems^8^.

In our previous study^10^, we characterized the bacterial and fungal communities in the rhizosphere of apple trees under seven soil management treatments. These treatments included both organic and inorganic regimes and were applied under uniform environmental conditions within a single orchard.

The present study was conducted in the same orchard, using the same apple trees and identical soil management practices as described in our previous study^10^. However, the sampling was carried out in the following growing season (2023), allowing us to explore year-to-year variation and the recurrence of management-associated microbial patterns in the rhizosphere microbiome under consistent field conditions.

In this work, we hypothesized that, under the same agronomic and environmental conditions, the overall structure of the rhizosphere microbiome would exhibit recurring patterns across growing seasons, with management-specific microbial signatures consistently associated with specific soil treatments. We also anticipated that certain microbial taxa—particularly those involved in essential ecosystem functions—would persist across years, forming a putative core microbial community, while others might exhibit greater variability in response to management inputs or microenvironmental changes.

To test this hypothesis, we conducted high-throughput sequencing of 16S rRNA (bacteria) and ITS (fungi) amplicons, followed by comprehensive bioinformatic and statistical analyses. We assessed both alpha and beta diversity, taxonomic composition, and microbial co-occurrence networks. By doing so, this study provides new insights into the consistency and responsiveness of soil microbial communities under long-term, real-world agricultural soil management practices.

## Methods

The experiment was conducted in an orchard located at the Warsaw University of Life Sciences, Wilanów, Poland (N 52°9′36.1″, E 21°5′58.2″). The apple orchard (*Malus* × *domestica* Borkh. cultivar ‘Red Jonaprince’, with feathered maiden trees grafted on M.9 rootstock) was established on fertile alluvial soils. These soils are characterized by a sandy silt texture, slightly acidic reaction (pHₖCl 5.7), low salinity (0.25 mg NaCl/dm^3^), humus content of 2.3%, and a very high level of available Mg (19.3 mg/100 g soil), medium K (11.6 mg/100 g soil), and very low P (1.77 mg/100 g soil). A detailed characterization of the soil properties is presented in^10^.

The experiment was set up in 2017 using a randomized block design, as described by Bokszczanin et al.^10^. The following methods of soil management in 1 m wide rows of trees were compared: (1) Herbicide strips (HSs) were used as a control, for which a soil width of 1 m was sprayed with herbicide (glyphosate in the Roundup 360 SL formulation at a dose of 4 Lha^−1^) at the beginning of June and after harvesting the fruit at the beginning of October in each year of the experiment; (2) mechanical cultivation (MC), which was used in rows of trees 1 m wide using a rototiller-type tool mounted on the rear of a tractor equipped with a hydraulic system, enabling access to the area between trees, and the soil was tilled up to 10 cm deep up to six times during the growing period (from April to October) depending on weather conditions; (3) synthetic mulch (BC), for which a soil strip 1 m wide was mulched in rows of trees with a black polypropylene-woven ground cover of 100 g per m^2^ density; (4) organic litter (MM), for which a 1 m width of soil was mulched in rows of trees with a 10 cm layer of shredded straw from *Miscanthus* × *giganteus* (75 dm^3^ per tree); (5) organic litter (MMM), for which a soil width of 1 m and depth of 10 cm, before tree planting, was mixed in rows of trees with 75 dm^3^ of shredded straw from *M.* × *giganteus*, and directly after planting soil was mulched in rows of trees with a 10 cm layer of the straw; (6) organic litter (FM), for which a soil width of 1 m was mulched in rows of trees with a 10 cm layer of spent mushroom compost (75 dm^3^ per tree); and (7) organic litter (FMM), for which a soil width of 1 m and depth of 10 cm, before tree planting, was mixed in rows of trees with 75 dm^3^ of spent mushroom compost, and directly after planting soil was mulched in rows of trees with a 10 cm layer of the spent mushroom compost FM. All organic mulches were replenished during the trial to maintain a 10 cm layer.

Rhizosphere soil samples were collected in August 2023 using a sterile spade, near the stem and at depths of 10–40 cm, where the root system was denser. Soil samples were collected from each combination, replicated three times in plots of five trees. All samples were stored in sterile polythene bags and transported the short distance (approximately 7.5 km) back to the WULS laboratory, where they were immediately stored on ice in a cooling storage box for further processing within 24 h of sampling.

Soil physicochemical properties were determined for both 2020 and 2023 sampling points and included pH, available P, K, Mg, organic matter content, and salinity.

### DNA extraction and sequencing

Rhizosphere DNA was extracted from 0.5 g samples of each combination, replicated three times, using the DNeasy PowerSoil Kit (Qiagen, Hilden, Germany) prior to sequencing. A metagenomic analysis of the bacterial population was performed based on the hypervariable region V3-V4 of the 16S rRNA gene. The specific sequences of the 341F and 785R primers with adaptors, as well as the ITS1 primers used for fungal metagenomic analysis, are provided in the Supplementary Material Table. Further analysis was performed according to the procedure presented by Bokszczanin et al.^10^.

### Biological samples, 16S amplicon sequencing, and bioinformatic analysis

The 16S sequence processing bioinformatic pipeline was initiated using Illumina paired-end sequencing, targeting the V3-V4 region of the 16S rRNA gene. A rigorous initial quality assessment was conducted using FastQC (version 0.12.1) and MultiQC (version 1.23) tools to ensure the highest data quality. Following this, the sequences obtained were processed using the LotuS2 pipeline^11^. Quality filtering and read dereplication were implemented in sdm for the MiSeq Illumina platform, with a minimum sequence length of 210 bp instead of 200 bp. In LotuS2, the DADA2 (Divisive Amplicon Denoising Algorithm 2) algorithm^12^ was used to cluster sequences into ASVs (Amplicon Sequence Variants). Chimeras were detected and removed using reference-based and de novo chimera-checking algorithms in UCHIME3^13^, with the RDP reference database (rdp_gold.fa). ASVs were aligned by default against the phiX genome using Minimap2^14^, and those that produced significant matches were subsequently removed. The host contamination was also removed by mapping the reads against the reference *Malus domestica* Golden genome (Malus_domestica_golden.ASM211411v1.dna.toplevel.fa) (release 2024-07-13). Sequence clusters were curated and refined using the options for LULU (-lulu^15^. ASVs were aligned with Lambda v3.0.0^16^ to the SILVA 138.1 SSU database^17^ and KSGP^18^ for bacteria, and UNITE for fungi, to obtain taxonomic assignments for ASVs using the LotuS2 LCA (least common ancestor) algorithm with default parameters. ASVs that were assigned to categories such as *Eukaryota*, *Archaea*, mitochondria, and chloroplasts were included in the dataset. Unassigned taxonomic levels were assigned to the last known taxonomic level and sequentially numbered.

To minimize inflation of rare species in the community analysis, we removed ASVs that occurred fewer than 2 times in at least 10% of the samples.

### Statistical analysis

For selected analyses, treatments were grouped into “organic” (miscanthus-based mulches and spent mushroom compost) and “inorganic” (herbicide strip, mechanical cultivation, and synthetic mulch) categories to reflect the presence or absence of organic matter inputs.

Prior to statistical analysis, the raw count data underwent quality control and preprocessing. Taxa present in less than 10% of the samples were excluded to minimize the impact of rare taxa on the analysis. The count data were rarefied to address the compositional nature of microbiome or mycobiome data.

To explore potential interactions among microbial taxa, we conducted pairwise Pearson correlation analyses, which measure the linear relationship between two continuous variables. Given the large number of pairwise comparisons, controlling for false positives was essential. The Benjamini–Hochberg procedure was applied to adjust p-values for multiple testing using the p.adjust function in the base stats package. Adjusted *p*-values (q-values) less than 0.05 were considered statistically significant.

Significant correlations were used to construct microbial or fungal association networks, with nodes representing taxa and edges representing significant correlations between them. The igraph package was utilized for network construction and analysis. Edge weights were assigned based on the magnitude of the correlation coefficients. Network topology parameters, such as node degree, betweenness centrality, and clustering coefficients, were calculated to identify the network’s key taxa and community structures. Co-occurrence networks were constructed at an aggregated level (organic vs. inorganic) to reflect functional differences in soil management rather than mechanistic uniformity within groups. However, given the relatively small number of samples per subgroup, the resulting network structures should be interpreted with caution, particularly at the edge level.

Heatmaps for each maltreatment group were generated to display the relative abundance of the most dominant taxa across samples.

Heat tree analysis was utilized to visualize the differential abundance of taxa across hierarchical taxonomic classifications between the baseline and post-fasting groups. The analysis utilized Metacoder v.0.3.6^19^. The analysis focused on the genus level, providing detailed insights into microbial changes. The Reingold-Tilford algorithm was selected for tree layout to optimize the hierarchical representation of taxa. The analysis was set to comparison mode, specifically comparing the post-fasting and baseline control groups. Differences in taxa abundance between groups were assessed using the inparametric Wilcoxon rank-sum test at each taxonomic level. P-values were adjusted for multiple comparisons using the Benjamini–Hochberg false discovery rate correction. Each node in the heat tree visualization represents a taxon, and edges denote taxonomic relationships. Node sizes correspond to the mean relative abundance of taxa, while node colors reflect the log2-fold change between groups.

Microbial abundance data were processed using the microbiome package and managed as phyloseq objects using the phyloseq package v.1.22.3^20^. Data normalization and transformation steps were performed to account for compositional and sequencing-depth variations. The prevalence of each taxon across samples was calculated as the proportion of samples in which its abundance exceeded a specified detection threshold, using the prevalence() function from the microbiome R package. Raw counts were transformed to relative abundances (compositional scaling) with the transform (“compositional”) function, and only taxa meeting the predefined abundance criteria were retained for core analysis. Core taxa were then identified with the core_members() function, which selects taxa that satisfy both prevalence and abundance thresholds. To visualize the distribution of core taxa, a heatmap of prevalence and abundance across samples was generated using the plot core() function.

All statistical analyses were conducted using R v.4.3.2 and Python v.3.12 for Fedora Linux (v.41). Graphics were generated with ggplot2 v3.5.1^21^, plotly v5.24.1^22^, and seaborn v.0.13.2^23^.

Sequencing data from 2020 and 2023 were generated in separate runs and processed independently using the same bioinformatic pipeline. To avoid potential batch effects, direct statistical comparisons between years were not performed. Instead, results from each year were analyzed separately and juxtaposed to explore recurring patterns and similarities in microbial community composition across different soil management treatments. This approach allowed us to identify management-associated microbial signatures that persisted across years while accounting for potential inter-run variability.

## Results

The experiment revealed that soil physicochemical properties were largely stable between 2020 and 2023 across most cultivation systems (Table 1). No significant differences were observed in most treatments, although slight, non-significant increases in pH and phosphorus content were observed in several variants. In contrast, the spent mushroom compost treatments showed significant increases in pH, organic matter content, and nutrient levels compared with other treatments.

**Table 1:** Physicochemical soil properties (0–40 cm layer) in 2020 and 2023 across different soil management systems.

Soil management systems	pH	P	K	Mg	Organic matter	Salinity
	KCl	mg 100 g⁻^1^			%	g KCl L⁻^1^
*2020*						
Black cover material	5.7ab	0.8a	10.9a	15.3a	2.31ab	0.28a
Mechanical cultivation	5.2a	1.5a	15.2ab	15.0a	2.50ab	0.30a
Herbicide strip	5.2a	1.2a	18.3abc	15.5a	2.75ab	0.3a
Miscanthus 1	5.3a	1.2a	16.5abc	16.0a	2.60ab	0.28a
Miscanthus 2	5.3a	1.3a	13.1ab	17.0ab	2.96abc	0.28a
Mushroom 1	5.9abcd	34.7b	73.3d	28.8b	6.57d	2.14c
Mushroom 2	6.4bcd	23.9ab	74.6d	20.3ab	6.41d	2.19c
*2023*						
Black cover material	5.5a	2.0a	13.5ab	14.8a	2.11ab	0.37a
Mechanical cultivation	5.5a	2.1a	11.9ab	15.0a	2.29ab	0.32a
Herbicide strip	5.2a	2.1a	14.4ab	15.0a	1.24a	0.35a
Miscanthus 1	5.7abc	4.6ab	17.0abc	15.2a	2.48ab	0.35a
Miscanthus 2	5.5a	4.8ab	16.1abc	15.7a	2.79ab	0.33a
Mushroom 1	6.6 cd	32.4ab	30.4c	13.0a	5.05 cd	1.11b
Mushroom 2	6.7d	35.3b	26.3bc	12.8a	3.52bc	1.17b

The obtained results were verified statistically by means of Anova for two-factor experiments and the Tukey test. The programme *Statistica 10.0PL* was used for statistical analysis. Means followed by the same letter within column and do not differ significantly at *p* _ 0.05.

Values represent means (n = 3). Different lowercase letters within a column indicate statistically significant differences between treatments and years, as determined by the Tukey test (*p* < 0.05). Abbreviations: P – phosphorus, K – potassium, Mg – magnesium.

Overall, these results indicate that, aside from compost-amended plots, soil chemical properties remained relatively stable over time.

### Alpha and beta diversity

**Fig. 1:**
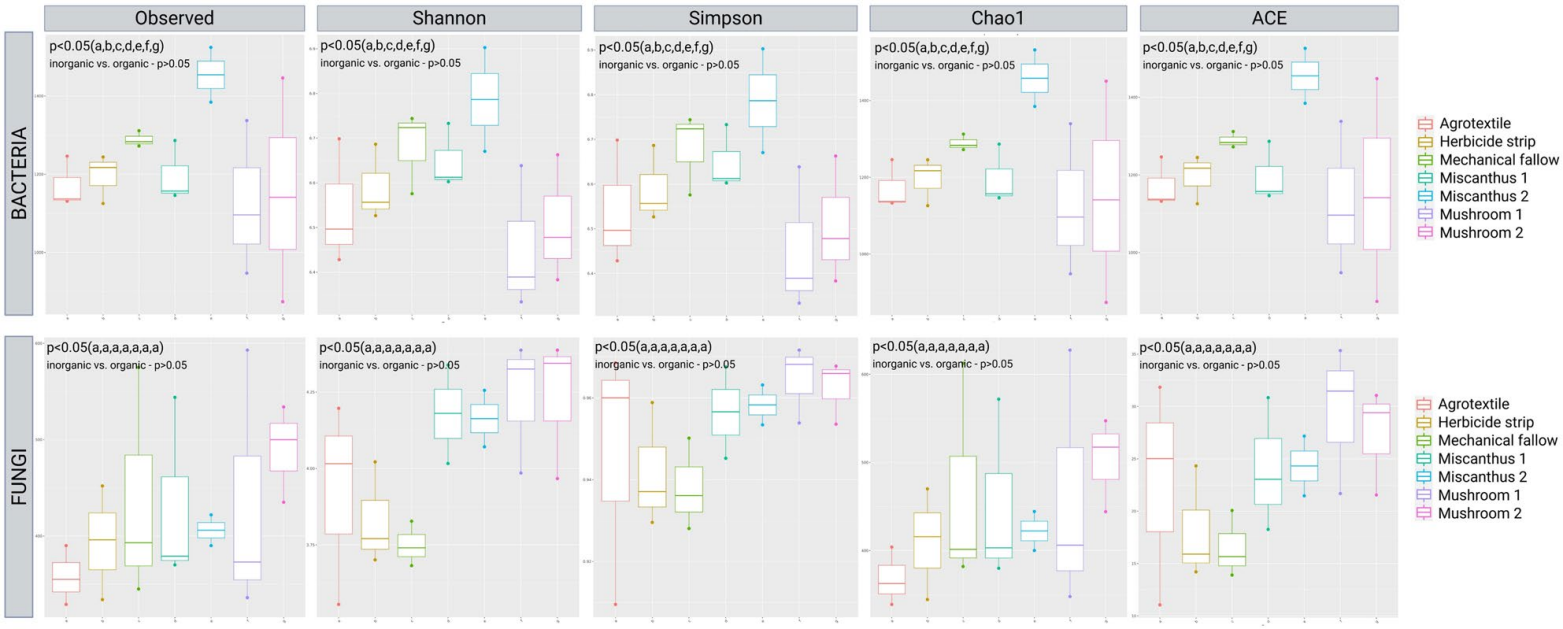
Alpha diversity of bacterial and fungal communities across management treatments. Boxplots show alpha-diversity indices for bacterial (top row) and fungal (bottom row) communities under different soil management treatments: agrotextile, herbicide strip, mechanical fallow, *Miscanthus* (two variants), and mushroom substrate (two variants). Diversity metrics include observed richness (Observed), Shannon diversity index (Shannon), Simpson diversity index (Simpson), Chao1 richness estimator (Chao1), and ACE richness estimator (ACE). Boxes represent the interquartile range (IQR; 25th–75th percentiles), horizontal lines within boxes indicate medians, and whiskers extend to the most extreme values within 1.5 × IQR. Points represent individual samples. Lowercase letters (**a**–**g**) denote statistically significant differences among treatments within each panel based on post hoc comparisons (*p* < 0.05); treatments sharing the same letter are not significantly different. The annotation “inorganic vs. organic – *p* > 0.05” indicates that there is no significant difference between these two broader management categories.

**Fig. 2:**
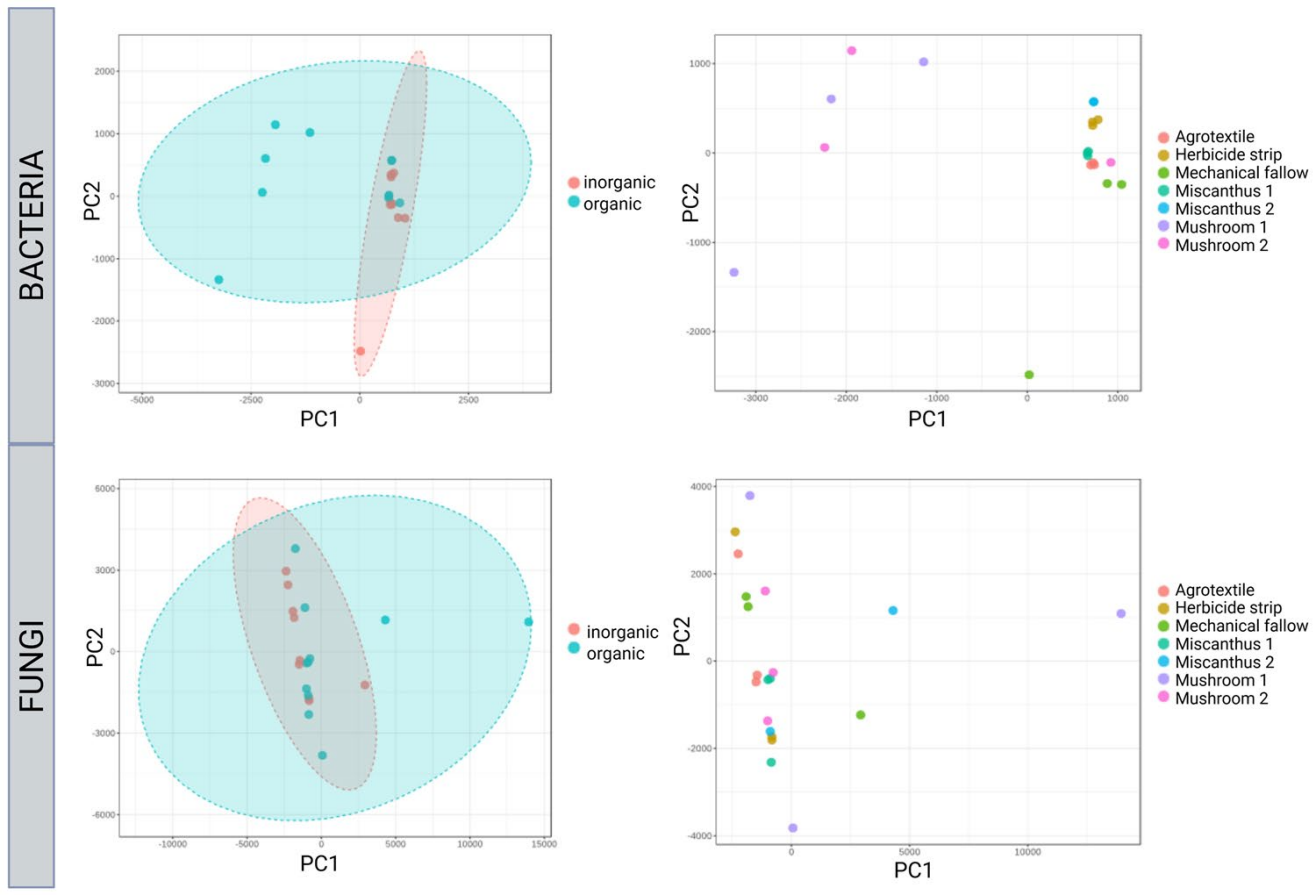
Beta diversity of bacterial and fungal communities under different soil management treatments. Principal coordinates analysis (PCoA) ordination plots illustrate differences in community composition for bacterial (top row) and fungal/eukaryotic (bottom row) communities. Axes (PC1 and PC2) represent the first two principal coordinates, with the percentage of total variation explained indicated on each axis. The left panels show comparisons between broad management categories: organic (MM, MMM, FM, FMM) and inorganic (HS, MC, BC), with shaded ellipses representing 95% confidence intervals around group centroids. Right panels display the seven individual mulching treatments. Statistical significance was assessed via PERMANOVA: Bacteria: individual treatments (F = 5.55;R2 = 0.72;*p* = 0.00); organic vs. inorganic (F = 4.91;R2 = 0.21;*p* = 0.01); Fungi/Eukaryotes: individual treatments (F = 1.41;R2 = 0.39;*p* = 0.056); organic vs. inorganic (F = 2.54;R2 = 0.12;*p* = 0.01). The spatial separation between points reflects dissimilarity in microbial community composition, with greater distances indicating more distinct communities.

In the alpha diversity analyses for bacteria (Fig. 1), all six metrics (Observed, Shannon, Simpson, Chao1, and ACE) revealed significant differences among the seven soil management treatments (Agrotextile, Herbicide strip, Mechanical fallow, Miscanthus 1, Miscanthus 2, Mushroom 1, and Mushroom 2). Kruskal–Wallis tests for Observed indices both yielded *p*-values of 0.01, while the Shannon index had a *p*-value = 0.02, the Simpson index had a *p*-value = 0.018, the Chao1 index had a *p*-value = 0.020, and the ACE index had a *p*-value = 0.02 (statistic: 15.138). These findings indicate that both richness-based (Observed, Chao1, ACE) and diversity-based (Shannon, Simpson) metrics differed among the treatments. However, when samples were aggregated into two broader categories, inorganic and organic methods, alpha diversity was not significantly different (*p*-values close to 1.0), suggesting that this binary classification captures only part of the variability and may mask differences due to high heterogeneity within the inorganic category. In contrast, beta-diversity analyses of bacterial communities (Fig. 2) revealed clear differences in community composition. Principal component analysis (PCA) followed by PERMANOVA revealed significant differences among the seven management practices (F-value = 5.55; R-squared = 0.72; *p*-value = 0.00), indicating that the specific treatment explained a substantial proportion of variance in community structure. When the data were consolidated into the two general categories of inorganic versus organic methods, PERMANOVA still detected significant differences in beta diversity (F-value = 4.91; R-squared = 0.21; *p*-value = 0.01), showing that, while the reduction to two groups obscured differences in alpha diversity, it did not eliminate compositional distinctions at the community level.

The alpha diversity of fungi-associated communities, assessed using Observed, Shannon, Simpson, Chao1, and ACE indices, did not differ significantly across the seven soil management conditions (Agrotextile, Herbicide strip, Mechanical fallow, Miscanthus 1, Miscanthus 2, Mushroom 1, and Mushroom 2) (Fig. 1). The Kruskal–Wallis tests yielded p-values ranging from 0.22 (Simpson) to 0.33 (ACE), indicating no detectable effects on species richness or diversity metrics. Consistent with these results, when the same data were grouped into inorganic and organic management methods, no meaningful differences in alpha diversity were observed (*p*-values close to 1.0).

In contrast, the beta diversity analysis, based on a PCA and PERMANOVA, revealed that the broader classification into inorganic and organic methods produced a significant difference in community composition (F = 2.54; R^2^ = 0.12; *p* = 0.01), suggesting that, despite the absence of variation in species richness or evenness, the overall structure of the communities differs between these two broad groups (Fig. 2). However, when the original seven management conditions were included in a single model, the PERMANOVA was marginally insignificant (F = 1.4156; R^2^ = 0.39517; *p* = 0.056), indicating that, while some compositional trends may exist across the full set of conditions, these differences did not reach the conventional threshold for significance. Together, these findings suggest that fungal alpha-diversity measures may be relatively insensitive to the soil management practices examined here. Conversely, certain compositional shifts detectable by beta diversity analysis are more evident when contrasting inorganic versus organic treatments (Fig. 1). This contrast underscores that the general management strategy is a more robust driver of fungal community composition than the specific variations between individual mulching types, thereby validating the decision to aggregate treatments for high-level functional comparisons.

### Community profiling

**Fig. 3:**
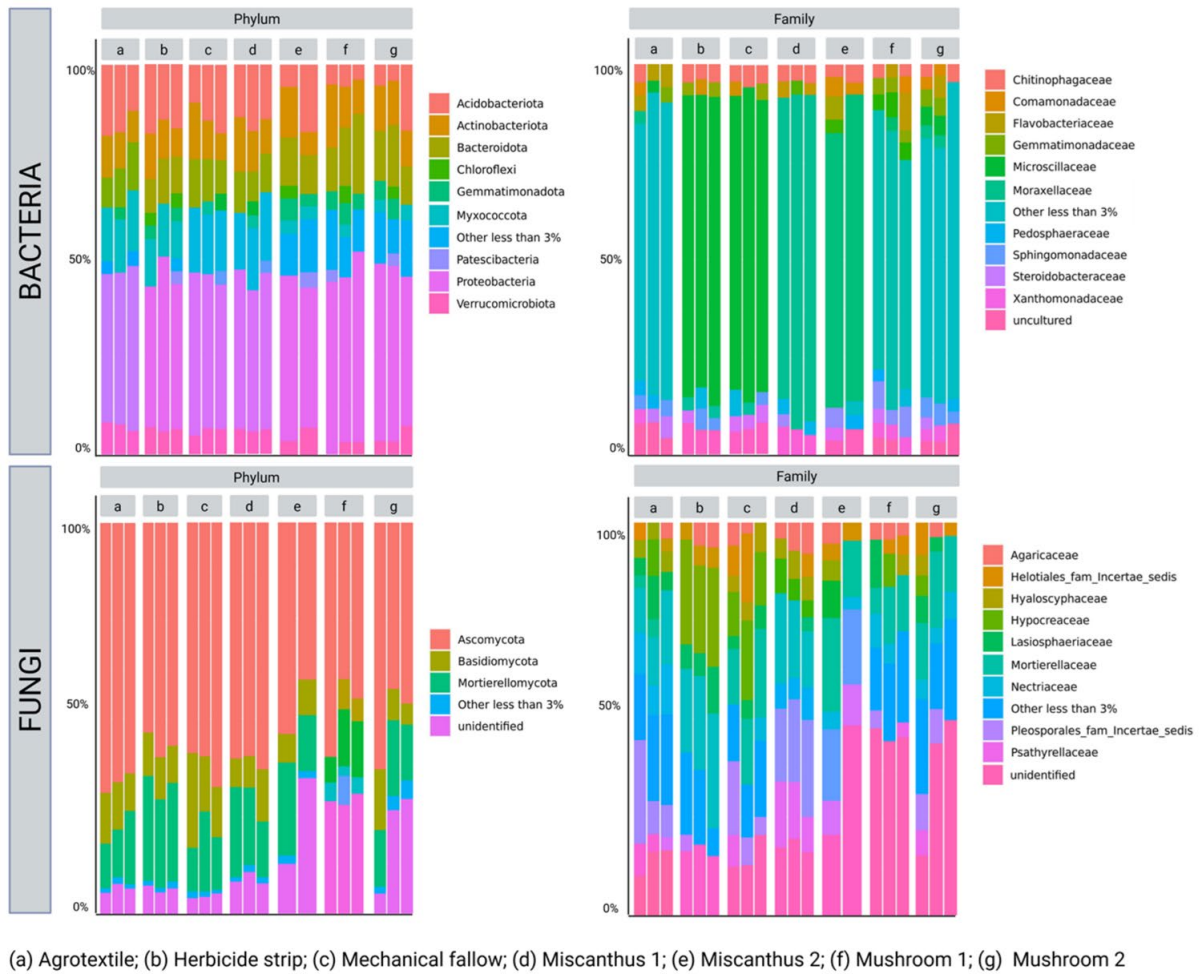
Relative abundance of bacterial and fungal communities at phylum and family levels across treatments in 2023 year of the experiment. Stacked bar plots showing the relative abundance (%) of dominant bacterial (top) and fungal (bottom) taxa at the phylum (left) and family (right) levels across treatments. Letters (**a**–**g**) denote treatments: (**a**) agrotextile, (**b**) herbicide strip, (**c**) mechanical fallow, (**d**) *Miscanthus* 1, (e) *Miscanthus* 2, (**f**) mushroom 1, and (**g**) mushroom 2. Taxa with relative abundance < 3% are grouped as “Other less than 3%”.

Across inorganic and organic samples, and across all samples without grouping, *Pseudomonadota* and *Acidobacteriota* are the most abundant in all conditions (Fig. 3). *Bacteroidota*, *Actinobacteriota,* and *Verrucomicrobiota* are consistently present as secondary contributors. In the organic samples, the following phyla dominated: *Pseudomonadota*, *Bacteroidota*, *Actinobacteriota*, *Gemmatimonadota, Chloroflexi,* and *Firmicutes*. In inorganic samples encompassing Mechanical fallow, Agrotextile, and herbicide strip, there was a higher abundance of *Acidobacteriota*, *Verrucomicrobiota*, *Patescibacteria*, and *Armatimonadota* (Fig. 3).

In organic samples, the following families of bacteria were dominant in inorganic samples: *Sphingomonadaceae*, *Gemmatimonadaceae*, *Microscillaceae*, *Steroidobacteraceae*, *Streptomycetaceae*, *Flavobacteriaceae*, *Xanthomonadaceae*, and *Moraxellaceae*. In the inorganic treatments, the following prevailed: *Comamonadaceae*, *Rhodanobacteraceae*, *Xanthobacteraceae*, *Pedosphaeraceae*, *Pseudomonadaceae*, SC-I-84 (*Burkholderiales*), and *Nitrosomonadaceae* (Fig. 3).

Because the datasets from 2020 and 2023 were generated and processed in separate sequencing runs, direct quantitative comparisons between years were avoided due to potential batch effects. Instead, the results were juxtaposed from both years to examine whether management-specific patterns in microbial communities were recurring. Analysis of bacterial family distribution revealed consistent differences between management systems across both study years. Families such as *Sphingomonadaceae*, *Xanthomonadaceae*, *Microscillaceae*, *Flavobacteriaceae*, and *Moraxellaceae* were consistently more abundant in organic soil management systems in both years. In contrast, the families *Chitinophagaceae*, *Xanthobacteraceae*, and *Acidobacteriales*_f_uncultured were enriched under inorganic practices across both years.

Some bacterial families exhibited temporal shifts in their association with management systems. The *Gemmatimonadaceae* were more abundant under inorganic management in 2020 but shifted to higher abundance in organic soils by 2023. In contrast, *Rhodanobacteraceae* and Pseudomonadaceae were more abundant in organic soils in 2020 but became more dominant in inorganic soils in 2023. The opposite trend was observed for *Nitrosomonadaceae*, which showed higher abundance in inorganic soils in 2020 but was enriched in organic soils by 2023 (Fig. 3).

Relative abundance profiles of eukaryotic taxa revealed clear differences between organic and inorganic soil systems (Fig. 3). Across all samples, *Ascomycota* dominated the communities in both management types, forming the majority of the relative abundance. *Mortierellomycota* and *Basidiomycota* were consistently present as secondary taxa, indicating a shared core eukaryotic microbiome. All *Ascomycota*, *Mortierellomycota, and Basidiomycota* were more abundant in inorganic samples than in organic samples*.*

Organic soils exhibited a pronounced enrichment of *Oomycota* and *Ciliophora* compared to inorganic treatments. In contrast, inorganic soils displayed higher abundances of *Ascomycota*, *Mortierellomycota,* and *Basidiomycota*. The conserved dominance of *Ascomycota* and *Basidiomycota* across both systems supports the presence of functionally important, management-independent taxa. In organic soil management systems, fungi families prevailed: unidentified family belonging to *Pleosporales* (*Dothideomycetes*), *Lasiosphaeriaceae* (*Sordariomycetes*), *Chaetomiaceae* (*Sordariales*), and *Microascaceae* (*Sordariomycetes*) (Fig. 3).

**Fig. 4:**
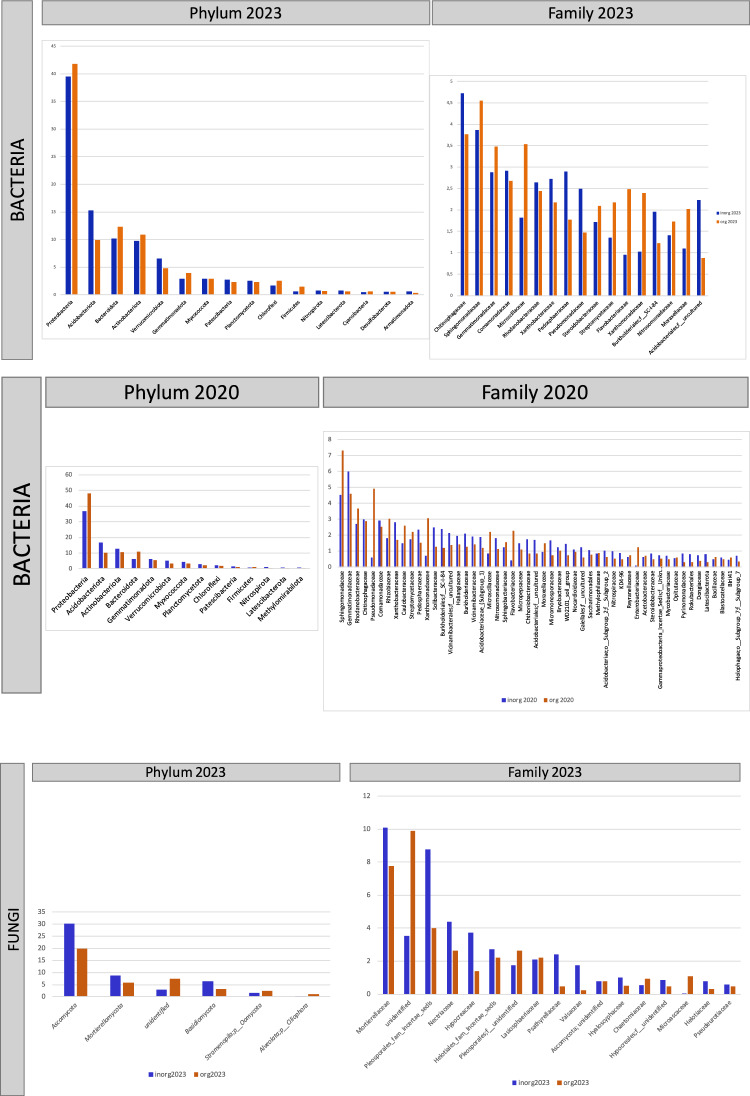

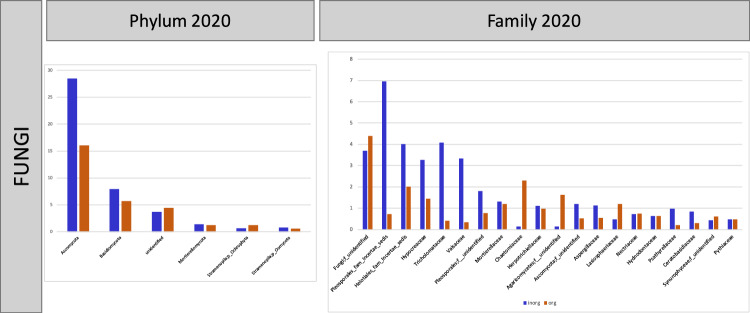
Juxtaposition of microbial community composition and recurring taxonomic patterns across the 2020 and 2023 growing seasons. Stacked bar plots [or heatmaps] illustrating the relative abundance of dominant bacterial (top) and fungal/eukaryotic (bottom) families under seven soil management regimes. Data from separate sequencing runs (2020 and 2023) are juxtaposed to identify persistent, management-specific microbial signatures while accounting for potential inter-run batch effects. The visualization highlights recurring associations, such as the consistent enrichment of *Sphingomonadaceae* and *Flavobacteriaceae* in organic systems and *Mortierellaceae* and *Comamonadaceae* in inorganic systems across both study years. Detailed results for 2020 were previously described in Bokszczanin et al.^10^.

Between 2020 and 2023, the composition of soil fungal communities under organic and inorganic mulching showed notable shifts in taxonomic abundance. In 2023, the most abundant families were *Mortierellaceae*, unidentified taxa, and *Pleosporales_fam. Incertae sedis*. *Mortierellaceae* were consistently more abundant in soils under inorganic mulching, with the difference being more pronounced in 2023 than in 2020. Similarly, *Pleosporales_fam. Incertae sedis*, *Hypocreaceae*, *Helotiales_fam. Incertae sedis*, and *Valsaceae* were more abundant in the inorganic treatment in both years. *Nectriaceae* showed comparable levels between treatments in 2020, but were more abundant in inorganic treated soils in 2023. By contrast, *Lasiosphaeriaceae* and *Chaetomiaceae* were relatively enriched under organic mulching, though the difference was stronger in 2020 and only marginal in 2023 (Fig. 4).

In 2020, the community structure was dominated by unidentified fungi, *Pleosporales_fam. Incertae sedis*, and *Helotiales_fam. Incertae sedis*, followed by *Hypocreaceae*, *Tricholomataceae*, and *Valsaceae*. *Mortierellaceae* were less abundant in 2020 compared to 2023, but still occurred more frequently in inorganic mulch soils. Families such as *Herpotrichiellaceae*, *Aspergillaceae*, *Hydnodontaceae*, and *Ceratobasidiaceae* were detected only among the dominant groups in 2020, while their abundance was negligible or undetected in 2023. Overall, the data indicate that inorganic mulching consistently favored the proliferation of several fungal families (e.g., *Mortierellaceae*, *Hypocreaceae*, *Valsaceae*), whereas organic mulching was more closely associated with the enrichment of saprotrophic groups, particularly *Lasiosphaeriaceae* and *Chaetomiaceae*, particularly in 2020.

**Fig. 5:**
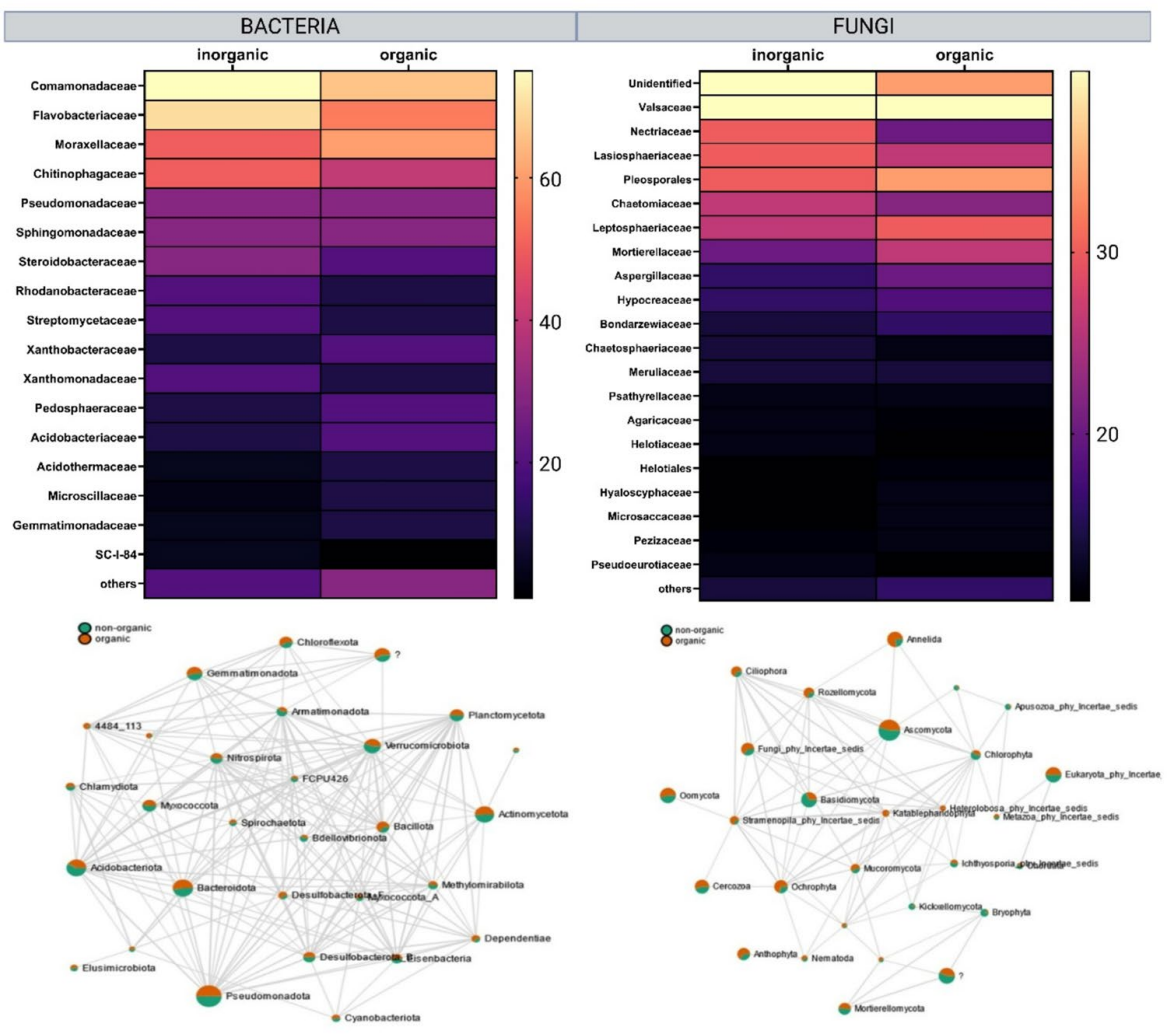
Comparative analysis of microbial community structure and co-occurrence networks between management regimes. (Top) Heatmaps representing the relative abundance of dominant bacterial (left) and fungal (right) families across the aggregated organic and inorganic management systems. Color intensity reflects the mean relative abundance within each group. (Bottom) Co-occurrence networks showing the ecological interactions within bacterial and fungal/eukaryotic communities. Nodes represent individual taxa (phylum level shown by color), and edges represent significant Pearson correlations (*p* < 0.05, Benjamini–Hochberg adjusted). The bacterial network (left) shows a densely interconnected core, while the fungal/eukaryotic network (right) shows higher modularity and treatment-specific associations among peripheral taxa.

The heat maps reveal distinct differences in soil microbial community composition between inorganic and organic soil management systems. At the bacterial level, *Comamonadaceae*, *Flavobacteriaceae*, and *Moraxellaceae* were dominant across both systems. *Comamonadaceae* and *Flavobacteriaceae* were more prevalent under inorganic management, whereas *Moraxellaceae* were enriched under organic mulching (Fig. 5).

For fungal communities, inorganic mulching was associated with higher abundances of *Valsaceae*, *Nectriaceae*, and *Lasiosphaeriaceae*, while organic mulching favored *Valsaceae*, *Pleosporales*, *Leptospheriaceae*, and *Mortierellaceae*. Notably, unidentified fungal taxa remained dominant in both treatments, though their relative contributions varied. In particular, *Pleosporales* were more abundant under organic than inorganic management (Fig. 5). Notably, while certain families such as *Comamonadaceae* and *Flavobacteriaceae* maintain high relative abundance across both management regimes, their specific prevalence and the topology of their associations within the co-occurrence networks differ significantly. This suggests that the management system influences not only the abundance of these core taxa but also their functional integration and ecological connectivity within the soil microbiom.

The co-occurrence network analysis revealed distinct structural patterns between bacterial and fungal/eukaryotic communities under organic and inorganic soil management systems. The bacterial network was densely interconnected, with several large, centrally positioned hub taxa (e.g., *Pseudomonadota*, *Actinomycetota*, *Acidobacteriota*, *Bacteroidota*) shared across both treatments, suggesting a stable bacterial core. In contrast, the fungal/eukaryotic network displayed a more modular structure, with prominent hubs such as *Ascomycota* connecting to multiple sub-clusters, and peripheral nodes showing stronger associations with either organic or inorganic mulching. For example, *Annelida* appeared more associated with organic soils, while *Mortierellomycota* and *Chlorophyta* were relatively enriched under inorganic conditions. Overall, these results indicate that while both networks retain a shared set of core taxa, mulching type influenced the distribution of peripheral and treatment-enriched groups, particularly within the fungal/eukaryotic assemblages (Fig. 5).

Eukaryotic Community Composition Is Significantly Affected by Management Regime.

Network-based differential abundance analysis (Fig. 5) revealed pronounced shifts in eukaryotic community composition between organic and inorganic soils. In organic soils, there was a significantly higher representation of *Ciliophora*, *Ochrophyta*, *Rozellomycota*, *Fungi phy. incertae sedis*, *Stramenopila phy. incertae sedis*, *Katablepharidophyta*, and *Heterolobosa phy. incertae sedis*.

In contrast, inorganic soils were enriched in *Basidiomycota*, *Chlorophyta*, *Kickxellomycota*, and *Zoopagomycota*, as indicated by negative log2 median ratios (blue edges). These taxa—especially *Basidiomycota*—were not only more abundant but also occupied central network positions, suggesting potential dominance or ecological connectivity under inorganic conditions.

**Fig. 6:**
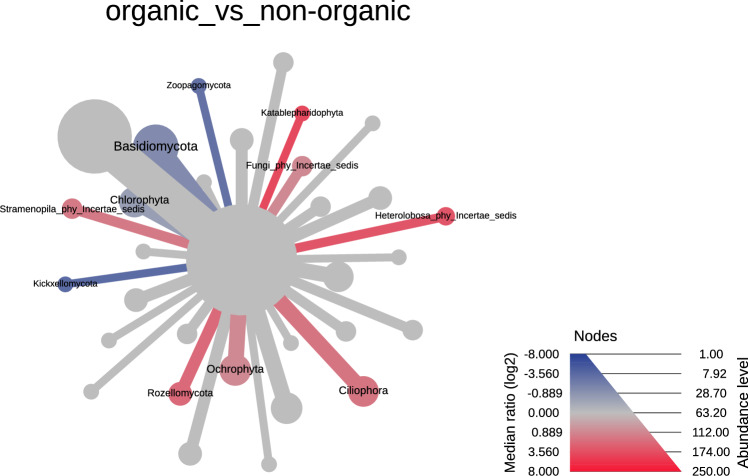
Heat tree visualization of differential taxonomic abundance in fungal and eukaryotic communities. The tree illustrates the hierarchical classification of taxa from phylum down to the genus level, comparing organic and inorganic soil management regimes. Node size corresponds to the mean relative abundance of each taxon, while node color reflects the log2-fold change between the two management groups. Taxa significantly enriched in organic systems are indicated by green nodes/edges, whereas those enriched in inorganic systems are shown in blue (Wilcoxon rank-sum test, Benjamini–Hochberg adjusted *p* < 0.05). This visualization highlights the enrichment of specific saprotrophic and eukaryotic groups (e.g., *Ciliophora*, *Ochrophyta*) in organic soils, compared with the prevalence of groups such as *Basidiomycota* and *Chlorophyta* in inorganic systems.

The heat tree visualization (Fig. 6) provided a hierarchical overview of the taxonomic shifts within the eukaryotic communities, comparing organic and inorganic soil management regimes from the phylum down to the genus level. This analysis revealed pronounced differences in community composition, highlighting the enrichment of specific lineages under different mulching strategies. Soils under organic management exhibited a significantly higher representation of a wide array of eukaryotic groups. Notable enrichments included *Ciliophora* (ciliates), *Ochrophyta*, *Rozellomycota*, *Katablepharidophyta*, and *Heterolobosa phy. incertae sedis*. Additionally, organic systems favored specific groups of *Oomycota* and saprotrophic fungi, as well as unclassified lineages of *Fungi* and *Stramenopila*. In contrast, inorganic soil management (including herbicide strips, mechanical cultivation, and agrotextile) was associated with an enrichment of *Basidiomycota*, *Chlorophyta* (green algae), *Kickxellomycota*, and *Zoopagomycota*. Specifically, members of the *Basidiomycota* not only showed higher relative abundance but also occupied central positions in the hierarchical network, suggesting their potential dominance or high ecological connectivity under these conditions. These findings demonstrate that the management regime—specifically the presence or absence of organic matter inputs—strongly influences the entire eukaryotic assemblage, promoting a more diverse array of saprotrophic and protozoan groups in organically amended soils.

## Discussion

The inclusion of soil physicochemical data from 2023 provides important context for interpreting the observed microbial community patterns. For the majority of treatments, soil properties remained stable between 2020 and 2023, with only minor, statistically nonsignificant changes observed. This suggests that, in these cases, differences in microbial communities are more likely associated with management practices and their direct ecological effects, rather than large shifts in measured soil physicochemical parameters.

In contrast, treatments amended with spent mushroom compost exhibited pronounced changes in soil properties, including increased pH, organic matter content, and nutrient availability. These changes are inherent to organic amendments and are likely to contribute to the observed shifts in microbial community structure. Therefore, microbial responses in these treatments should be interpreted as reflecting both the direct effects of organic inputs and the associated modification of soil physicochemical conditions.

Interestingly, not all organic amendments resulted in detectable changes in soil physicochemical properties. While spent mushroom compost significantly altered soil pH, nutrient availability, and organic matter content, miscanthus straw did not induce statistically significant changes in these parameters over the study period. This suggests that not all organic inputs exert equivalent effects on soil properties and microbial communities, and that the type and decomposability of organic material may play a key role.

Based on the 2023 dataset, alpha diversity did not differ significantly between the organic/inorganic groups. Groupings of the aggregated “organic” and “inorganic” categories were introduced to reflect a broad functional distinction between systems that supply organic matter to the soil and those that do not, a distinction particularly relevant to soil regeneration in orchard systems. However, the treatments within the “inorganic” category differ in their mode of action, including chemical (herbicide), mechanical disturbance, and physical soil cover. This heterogeneity may increase within-group variability and potentially mask differences in alpha diversity when analyzed at the aggregated level.

In contrast, treatments involving spent mushroom compost exerted a strong influence on both soil properties and microbial communities, and likely drive a substantial portion of the observed differences in beta diversity. Therefore, results from grouped analyses should be interpreted as indicative of general trends associated with organic matter inputs rather than uniform responses across all treatments. Soil treatment-level analyses provide a more detailed view of individual management effects and should be considered alongside grouped comparisons.

Beta diversity (PCA + PERMANOVA) revealed big differences in community structure both among the seven treatments and between the organic and inorganic groups.

This suggests that even if species richness remains constant, the identity and relative abundances of taxa can shift markedly between management types. Similar patterns have been documented in long-term pasture systems, where organic management was associated with greater bacterial richness and diversity than in inorganic soils, and community structure differed significantly between the two systems^24^. In grassland systems, the interactive effects of management practices (including organic vs inorganic) significantly influenced microbial beta diversity^25^.

In contrast, fungal alpha diversity did not vary significantly across either the seven treatments or the binary categories. This may reflect the greater resilience or functional redundancy of soil fungal communities. Fungi’s ability to form long-lived mycelial networks possibly buffers them from short-term environmental changes. Many soil fungi are saprotrophic generalists, capable of thriving across a broad range of organic substrates. Moreover, fungal communities are known to be more stable than the bacterial ones, potentially due to the longer lifespan of fungal cells and common spore formation^26^. Similar results were obtained by Pylak et al.^27^ in studies on α-diversity and β-diversity analyses of fungal microbial communities. Fungal communities, which are more stable, might help maintain soil health and plant resilience^27^. The stability of fungal communities and the variability in bacterial communities highlight the dynamic nature of soil microbiomes and their potential impact on apple cultivation.

Interestingly, although fungal alpha diversity remained stable, fungal beta diversity differed significantly between organic and inorganic treatments (*p* = 0.009), but not across all seven treatments (*p* = 0.056). This pattern suggests that broad-scale management regimes (e.g., synthetic inputs vs. organic matter amendments) may exert a more consistent influence on fungal community composition than finer-scale treatment differences, which could be partially obscured by temporal or spatial variability.

This interpretation is consistent with previous studies showing that agricultural intensification tends to reduce soil microbial biodiversity and simplify co-occurrence networks, resulting in fewer keystone taxa, whereas organic or low-input systems are often associated with more complex networks and a higher prevalence of mycorrhizal fungi among keystone taxa. These shifts in network organization have been linked to changes in ecosystem functioning^28^.

In line with this, our results are also consistent with findings by Lai et al.^29^, who demonstrated that β-diversity (community structure) of bacterial and fungal communities is a stronger predictor of ecosystem functions than alpha diversity alone.

Co-occurrence network inference is known to be sensitive to sample size. In the present study, networks were constructed from a relatively limited number of samples per subgroup (organic vs. inorganic), which may increase the likelihood of spurious correlations and affect the stability of inferred associations. Accordingly, the network structures are interpreted primarily at the level of general topological patterns rather than as evidence of specific pairwise interactions. Despite this limitation, consistent differences in overall network organization between management types were observed, suggesting that the detected patterns may reflect underlying ecological structuring rather than purely methodological artifacts.

A recent synthesis reports that organic farming increases microbial abundance and activity, thereby enhancing soil carbon (C) and nitrogen (N) levels, which helps explain why organically managed soils often support richer, more functionally diverse microbiomes^30^. Although soil organic carbon (C-org) is fundamental to soil health, the study by Olchowik et al.^31^ suggests that under extreme urban conditions, the effect of carbon on fungal species richness may be masked by more immediate stress factors such as salinity and alkalization. In these studies, a high organic matter content was found to be significantly associated with the abundance of colonized root tips and the number of symbiotic fungal species. However, species richness was primarily influenced by soil chemical characteristics, which were shaped by urban transformation. Interestingly, the effect of this transformation on overall diversity was not negative; rather, it led to a differentiation of taxonomic composition among the analyzed clusters^31^. In general Organic management consistently enhances soil microbial network complexity, functional diversity, and nutrient cycling efficiency^32,33^. Non-organic or conventional systems, particularly when land use shifts, can promote dominance of fungal groups such as Ascomycota and Basidiomycota, potentially reshaping network structure^34^.

*Pseudomonadota* and *Acidobacteriota* were the most abundant phyla across all treatments, while secondary contributors included *Bacteroidota*, *Actinobacteriota*, and *Verrucomicrobiota*.

Here, we observed that in organic samples, *Pseudomonadota*, *Bacteroidota*, *Actinomycetota*, and *Chloroflexi* dominated in both the present study and analogous studies from 2020^10^. Li et al.^35^ concluded that organic farming was associated with a higher relative abundance of *Pseudomonadota*, while *Actinobacteriota* and *Chloroflexi* were more abundant in conventional farming. Shen and Lin^36^ observed that green manure treatment altered bacterial communities from oligotrophic (Acidobacteria and *Chloroflexi*) to copiotrophic (*Bacteroidetes* and *Pseudomonadota*). Wang et al.^37^ found that *Pseudomonadota*, *Actinobacteriota*, and *Acidobacteria* were the main bacterial groups in the apple root zone soil, while *Pseudomonadota* and *Actinobacteriota* were found in the rhizosphere soil, and *Pseudomonadota* and Bacteroidetes were found in the root.

In our research on the inorganic samples from our experiment, the phyla *Acidobacteriota*, *Verrucomicrobiota*, *Armatimonadota*, *Methylomirabilota*, and *Elusimicrobiota* predominated over the organic samples, and this pattern remained consistent over the years^10^. Particularly important are *Acidobacteriota*, *Verrucomicrobiota*, and *Armatimonadota*, which play roles in the decomposition of complex organic compounds, nutrient availability, humus formation, soil structure development, and indirectly in shaping the soil’s water–air properties^38,39^. However, species belonging to *Verrucomicrobiota* and *Armatimonadota* are often found in soils with poor water–air and nutrient conditions, high salinity, and low pH^40–42^.

Here, organic mulching favored bacterial families associated with organic matter turnover and decomposition: *Sphingomonadaceae*, *Gemmatimonadaceae*, *Microscillaceae*, *Steroidobacteraceae*, *Streptomycetaceae*, *Flavobacteriaceae*, *Xanthomonadaceae*, and *Moraxellaceae*. The *Sphingomonadaceae* species can utilize a wide range of organic compounds and grow and survive under low-nutrient conditions^43^. *Sphingomonadaceae* secrete exopolysaccharides to break chlamydospore dormancy^44^. For instance, rhizosphere microbiome analysis of high-susceptibility (HS) and low-susceptibility (LS) rice varieties revealed that HS varieties recruited bacteria from the *Sphingomonadaceae* family, thereby facilitating the breakdown of chlamydospore dormancy through secreted exopolysaccharides^44^. Moreover, certain studies have identified specific strains of *Sphingomonas* that enhance plant growth^45^ and mitigate abiotic stresses, including water stress, by driving root developmental plasticity and regulating plant physiology and metabolism^46^. Many *Flavobacterium* spp. are common plant-associated/rhizosphere bacteria that decompose complex polysaccharides, contribute to carbon cycling, and can act as plant-growth-promoting or biocontrol agents in some cases^47^. *Moraxellaceae* is responsible for denitrification and the degradation of organic pollutants^48^.

In contrast, in our study, inorganic mulching promoted families such as *Comamonadaceae*, *Rhodanobacteraceae*, *Xanthobacteraceae*, *Pedosphaeraceae*, *Pseudomonadaceae*, SC-I-84 (Burkholderiales), and *Nitrosomonadaceae*. *Comamonadaceae* are often abundant in soil and the rhizosphere; members include aerobic, metabolically versatile taxa involved in organic matter decomposition, nutrient cycling, and sometimes in the oxidation/transformations of contaminants (e.g., arsenite oxidizers and other biogeochemical roles)^33^.

By juxtaposing results from separate sequencing runs across years (2020 and 2023), it identified recurring microbial community patterns associated with soil management practices, providing insights into the consistency and responsiveness of orchard soil microbiomes without making direct quantitative cross-year comparisons. Here several bacterial families exhibited consistent enrichment patterns across both 2020 and 2023, reflecting recurring associations in community responses to soil management: *Sphingomonadaceae*, *Xanthomonadaceae*, *Microscillaceae*, *Flavobacteriaceae*, *Moraxellaceae* (organic) and *Chitinophagaceae*, *Xanthobacteraceae*, *Acidobacteriales*; f__uncultured (inorganic). Despite the lack of direct comparability between sequencing runs, the repeated detection of similar taxonomic associations with specific management regimes suggests that these patterns are biologically meaningful rather than purely technical artifacts. *Flavobacterium* is a genus within the phylum *Bacteroidota* that remains relatively unexplored. Recent analyses of plant microbiota have identified the phylum *Bacteroidota* as a major bacterial group in the plant rhizosphere. While *Flavobacterium* species within the phylum *Bacteroidota* have been recognized as pathogens in aquatic habitats, microbiome analysis and the characterization of novel Flavobacterium species have revealed their great diversity and potential presence in various environments. Many *Flavobacterium* species have made positive contributions to plant health and development, including promoting growth, controlling diseases, and enhancing tolerance to abiotic stress. The link between pectin, motility, and carbohydrate metabolism may be fundamental to flavobacterial rhizosphere competence^47,49^.

In our studies, some families showed temporal shifts across years, including *Gemmatimonadaceae*, *Rhodanobacteraceae*, *Pseudomonadaceae*, and *Nitrosomonadaceae*, suggesting that certain taxa may respond dynamically to interannual conditions.

In our research, specifically, *Microscillaceae* (*Bacteroidota*) and *Flavobacterium* sp. (*Bacteroidota*) were consistently more abundant in organic samples in both 2020 and 2023.

A particularly distinct population of microorganisms was observed in the variant with mushroom substrate. The positive effects of this compost on soil’s physical, chemical, and microbiological properties are well known^10,50–54^.

In our study, this cultivation system showed the highest proportion of the most functionally important groups of soil bacteria — *Pseudomonadota*, *Nitrosomonadaceae*, *Bacteroidota*, and *Gemmatimonadota*. These bacteria play a key role in the decomposition of organic matter and pollutants (biodegradation of pesticides, petroleum derivatives, and polycyclic aromatic hydrocarbons), in nutrient cycling (with *Pseudomonadota* being particularly important for atmospheric nitrogen fixation, nitrification, and denitrification), in humus formation, and in maintaining plant health^55–58^.

An increase in these bacterial groups in organically fertilized soils was also reported by Li et al.^35^ and Liu et al.^59^.

The core microbiome refers to a set of microorganisms (bacteria, fungi, etc.) that are consistently present and stable within a particular environment or host, despite variability in other microbial populations. Traditional methods for defining the core microbiome (such as Venn diagrams for comparing species lists) are overly simplistic and do not fully capture the biological roles of these microbes. More important than the exact species composition is the functional role of the microbiome—what the microorganisms do metabolically—which can be maintained even when the species composition changes^60^.

In our research, dominant fungal phyla across treatments included *Ascomycota*, *Mortierellomycota*, and *Basidiomycota*, forming a putative core microbiome observed across sampling years**.** *Ascomycota*, *Mortierellomycota*, and Basidiomycota were the main fungal groups in the apple root zone, rhizosphere, and root^61^. Previous studies have shown that these microbial communities are also the dominant communities in soil and plants^62,63^. Also Muñoz-Ramírez et al.^64^ found that the fungal communities featured Ascomycota, Mortierellomycota, and Basidiomycota. In the research performed by Ajeethan et al. ^65^, *Ascomycota*, *Basidiomycota*, *Mortierellomycota*, and *Glomeromycota* were the most relatively abundant fungal taxa in the apple orchard total microbiome.

Organic soils in our experiment were enriched in fungi associated with decomposition and organic matter turnover: *Lasiosphaeriaceae*, *Chaetomiaceae*, *Microascaceae*, and unidentified *Pleosporales* (*Dothideomycetes*). *Pleosporales *occur in various habitats as endophytes, epiphytes, or saprobes of dead plants, and are mainly involved in saprophylaxis and plant pathogenic processes ^66^. According to the microbial interaction theory, the intervention of pathogenic fungi leads to the existence of more probiotics than antagonists ^67^, which can explain the enrichment of anti-pathogenic beneficial bacteria in the apple rhizosphere of organic samples. Liu et al.^59^ identified *Pleosporales* as one of the most important fungal biomarkers for the bulk soil, and predicted a higher relative abundance of pathogenic fungi in bulk soil compared to rhizosphere soil. It should be emphasized that even within the rhizosphere fungal community, both species richness and the number of colonized root tips are strongly influenced by habitat-related factors. In the study by Olchowik et al.^31^, the vitality of the examined linden trees and the composition of ectomycorrhizal (ECM) species were found to be significantly associated with soil properties, particularly with heavy metal contamination. The results demonstrated a negative correlation between soil pH and salinity, as well as between both plant condition and ECM colonization, indicating that the deterioration of soil conditions is closely linked to declining tree vitality, reduced enzymatic activity of mycorrhizae, and a lower abundance of ECM fungi under increasing anthropogenic pressure.

Inorganic mulching favored families such as *Mortierellaceae*, *Valsaceae*, *Hypocreaceae*, *Helotiales*_fam. *Incertae sedis*, and *Nectriaceae*, with these patterns consistently observed in both sampling years. Temporal shifts were observed for some fungal families: *Lasiosphaeriaceae* and *Chaetomiaceae* were more enriched under organic mulching in 2020, whereas differences were less pronounced in 2023, reflecting modest interannual variation. *Mortierellaceae* are widespread saprotrophs in soil involved in decomposition, carbon cycling, and nutrient (including P) solubilisation; some *Mortierella* spp. have plant-growth-promoting properties^68^.

Co-occurrence networks revealed a densely interconnected bacterial core shared across treatments, whereas fungal/eukaryotic networks were more modular and sensitive to mulching type.

Peripheral fungal/eukaryotic taxa showed stronger associations with either organic or inorganic systems, e.g., *Annelida* enriched in organic soils and *Mortierellomycota* in inorganic soils.

Overall, both bacterial and fungal communities exhibit shared core taxa across sampling years; however, mulching type influences peripheral taxa and the functional groups involved in decomposition and nutrient cycling.

The research was conducted in an orchard setting, but the results also sound promising in the context of urban forests and the potential to improve the habitat conditions of newly planted and mature trees, thereby improving tree health and, as a result, the resilience of cities to climate change, which may be the basis for further research.

## Conclusions

Our results demonstrate that soil mulching management strongly influences microbial community composition, with distinct effects on both bacteria and fungi. Based on the 2023 dataset, bacterial alpha diversity differed significantly among the seven treatments, although aggregation into broader inorganic versus organic categories obscured these differences. In contrast, beta diversity consistently revealed compositional shifts among treatments and between organic and inorganic management regimes.

*Pseudomonadota* and *Acidobacteriota* dominated across all treatments, with organic mulching favoring bacterial families associated with organic matter turnover and decomposition, including *Sphingomonadaceae*, *Gemmatimonadaceae*, *Microscillaceae*, *Steroidobacteraceae*, *Streptomycetaceae*, *Flavobacteriaceae*, *Xanthomonadaceae*, and *Moraxellaceae*. Inorganic mulching, in turn, promoted families such as *Comamonadaceae*, *Rhodanobacteraceae*, *Xanthobacteraceae*, *Pedosphaeraceae*, *Pseudomonadaceae*, SC-I-84, and *Nitrosomonadaceae*.

Across both sampling years (2020 and 2023), several bacterial families showed consistent associations with specific management regimes, although these observations should be interpreted as indicative of recurring patterns rather than direct temporal comparisons. At the same time, some taxa exhibited interannual variation, suggesting that microbial responses to soil management may include both persistent and dynamic components.

Fungal alpha diversity was largely insensitive to mulching type, whereas beta diversity analyses indicated that organic versus inorganic management shapes community composition. Organic soils were enriched in saprotrophic and decomposition-associated families such as Lasiosphaeriaceae, *Chaetomiaceae*, *Microascaceae*, and unidentified *Pleosporales*, while inorganic soils favored *Mortierellaceae*, *Valsaceae*, *Hypocreaceae*, *Helotiales*_fam. Incertae sedis, and *Nectriaceae*.

Co-occurrence network analyses suggested that bacterial communities may form a relatively cohesive core structure shared across treatments, whereas fungal networks are more modular and responsive to management type. Overall, these results suggest that organic and inorganic mulching systems are associated with distinct microbial community configurations, particularly in relation to taxa involved in organic matter turnover.

These findings highlight the importance of long-term soil management in shaping rhizosphere microbiomes and support the role of organic amendments in promoting microbial groups associated with organic matter turnover and soil functioning.

## Supplementary Information

Supplementary Information.

## Data Availability

The datasets generated and analysed during the current study are available in the NCBI RSA repository, under the following link https://www.ncbi.nlm.nih.gov/sra/PRJNA1357309
