# Supplementary material for: Soil management strategies shape bacterial and eukaryotic community structure in organic and inorganic systems of Malus × domestica production
Source: Sci Rep. 2026 Apr 30;16:18548. doi: 10.1038/s41598-026-49450-x (PMC13270100; doi:10.1038/s41598-026-49450-x)
Supplement: Supplementary file 1 — Supplementary Information. [file 41598_2026_49450_MOESM1_ESM.docx]

Supplementary Table

The specific sequences of the 341F and 785R primers with adaptors. The primers contain Illumina adaptor sequence (in italics) and V3-V4 16S rRNA locus-specific sequence

| Primer | Sequence |
| --- | --- |
| 341F | 5′ *TCGTCGGCAGCGTCAGATGTGTATAAGAGACAG*CCTACGGGNGGCWGCAG |
| 785R | 5′ *GTCTCGTGGGCTCGGAGATGTGTATAAGAGACAG*GACTACHVGGGTATCTAATCC |

The specific sequences of the ITS1FI2 and 5.8S primers with adaptors. The primers contain Illumina adaptor sequence (in italics) and the ITS1 locus-specific sequence.

| Primer | Sequence |
| --- | --- |
| ITS1FI2 | 5’ *TCGTCGGCAGCGTCAGATGTGTATAAGAGACAG*GAACCWGCGGARGGATCA |
| 5.8S | 5’ *GTCTCGTGGGCTCGGAGATGTGTATAAGAGACAG*CGCTGCGTTCTTCATCG |
